# Prediction of Soil Pollution Risk Based on Machine Learning and SHAP Interpretable Models in the Nansi Lake, China

**DOI:** 10.3390/toxics13040278

**Published:** 2025-04-05

**Authors:** Min Wang, Ruilin Zhang, Beibei Yan, Chengyuan Song, Yang Lv, Hengyi Zhao

**Affiliations:** 1 College of Earth Science and Engineering, Shandong University of Science and Technology, Qingdao 266590, China; brightwangm@163.com (M.W.); m15264559734@163.com (C.S.); 202211040813@sdust.edu.cn (Y.L.); 13002771066@163.com (H.Z.); 2 Geophysical Prospecting and Surveying Team of Shandong Bureau of Coal Geological, Jinan 250014, China; ybbsdust@126.com

**Keywords:** soil pollution, Nansi Lake, machine learning, XGBoost, SHAP, heavy metals, pollution load index, potential ecological risk index, classification model

## Abstract

To assess and predict the Nansi Lake soil pollution risk, we evaluate the soil environmental quality in the Nansi Lake region using machine learning techniques, combined with the SHapley Additive exPlanations (SHAP) model for interpretability. The primary objective was to predict the level of soil pollution caused by heavy metals, incorporating the traditional Pollution Load Index (PLI) and Potential Ecological Risk Index (PERI) methods. Through the integration of statistical characteristics, PLI, and PERI evaluations, a new assessment method was created, categorizing soil pollution into “Class0—no risk”, “Class1—low risk”, and “Class2—high risk”. Various machine learning models, including Support Vector Machine (SVM), Decision Tree Classifier (DT), Random Forest (RF), and XGBoost, were employed to predict the soil quality based on these indices. XGBoost demonstrated the highest accuracy, achieving a prediction accuracy of 93%. SHAP analysis was further applied to explain the machine learning model and determined that the accumulation of key pollutants such as cadmium (Cd) and mercury (Hg) may significantly produce soil pollution risk, and targeted management needs to be developed for these pollution features.

## 1. Introduction

Nansi Lake is located in the southwest of Shandong Province. It is composed of Nanyang Lake, Dushan Lake, Zhaoyang Lake, and Weishan Lake. It is an important reservoir for the South-to-North Water Diversion Project. However, rapid urbanization and industrialization since the 1970s have led to significant ecological challenges. One of the main concerns is the heavy metal pollution in the lake’s sediments and soils, particularly cadmium (Cd) and lead (Pb). The sediments accumulate these pollutants, primarily through industrial runoff and agricultural activities, exacerbating the overall ecological risk [1]. Soil conditions within the Nansi Lake basin reveal a notable imbalance in nutrient distribution [2]. Additionally, soil pollution from heavy metals, alongside improper agricultural practices, has raised concerns about the sustainability of soil health and its ability to support local ecosystems. This pollution has not only degraded water quality but also disrupted the natural soil functions, further challenging the region’s ecological stability [3,4].

Soil pollution, particularly caused by heavy metals, is a critical environmental concern due to its impact on human health and ecosystems. Traditional methods, such as the Pollution Load Index (PLI), have been widely used for assessing the degree of contamination. The PLI method relies on comparing the concentration of pollutants with standard background levels to evaluate pollution severity. However, traditional approaches face limitations, particularly in terms of accuracy and the inability to handle complex datasets with missing values [5,6]. To address these challenges, machine learning (ML) techniques have emerged as powerful tools for improving prediction accuracy and managing incomplete data. Algorithms like Random Forest (RF) and Support Vector Machines (SVMs) are increasingly employed for soil pollution prediction, as they can integrate large datasets and identify hidden patterns within complex environmental data [7,8]. Additionally, the application of machine learning techniques, such as the eXtreme Gradient Boosting (XGBoost) model, has shown significant improvements in forecasting soil contamination levels, especially in areas with missing or sparse data [9,10,11,12]

However, traditional methods mostly focus on analyzing heavy metals in the soil. We combined traditional pollution indices with ML models, and more reliable and effective strategies for soil pollution prediction can be developed, providing valuable insights for environmental monitoring.

## 2. Materials and Methods

### 2.1. Study Area

Nansi Lake, located in the southwestern region of Shandong Province, China, is a key freshwater reservoir within the Yellow River Basin. Positioned between from 34°40′ to 35°20′ N latitude and from 116°40′ to 117°10′ E longitude, it covers an area of approximately 1300 square kilometers. Nansi Lake is a key water body in the region, composed of four interconnected lakes: Weishan Lake, Dushan Lake, Zhaoyang Lake, and Nanyang Lake. As the largest freshwater lake in northern China, it plays a crucial role in regional water resource management, including water storage and flood control. The lake is integral to the South-to-North Water Diversion Project, serving as a vital storage reservoir for water supply. Nansi Lake also supports a range of agricultural, industrial, and ecological functions, making it an important area for both local livelihoods and regional development. Its strategic significance is further underscored by its location in the Huaihe River basin, one of the country’s key water source areas [13,14]. However, Nansi Lake faces growing ecological challenges, including water pollution and sediment contamination, which impact its water quality and ecosystem stability [15].

### 2.2. Sample Collection and Processing

**Figure 1:**
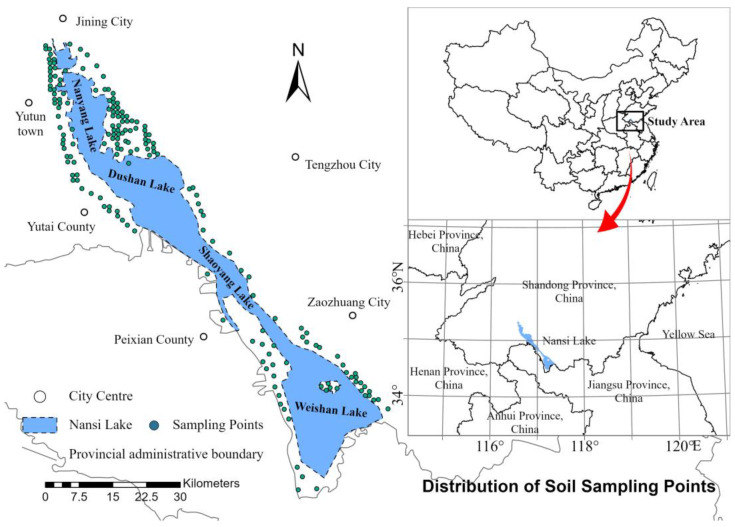
Spatial distribution map of sampling points.

In March 2024, a total of 180 soil samples were collected from the soil around the lake area of Nanshi Lake, and the distribution of all the samples is shown in Figure 1. Soil samples were collected following the guidelines in The Technical Specification for Soil Environmental Monitoring (HJ/T 166-2004) [16], and the sampling points were used to surround the Nansi Lake area in accordance with the block random sampling principle. After air-drying, the samples were sieved using a nylon sieve (10 mesh). According to the Specification of Land Quality Geochemical Assessment (DZ/T 0295-2016) and Technical Guideline for Site Soil and Groundwater Sampling of Volatile Organic Compounds (HJ 1019-2019) [17,18], the soil samples were stored in 60 mL soil sampling bottles. The samples were then sent to the Geological Laboratory of Shandong Provincial Coalfield Geological Bureau for further analysis, covering a total of 25 pollution features.

The pH of the samples was measured using the method outlined in Soil—Determination of pH—Potentiometry (HJ 962-2018) [19]. The concentrations of Cd, Cr, Cu, Co, Ni, Pb, Zn, Mn, Mo, and V were determined using Soil and Sediment—Determination of 19 Total Metal Elements—Inductively Coupled Plasma Mass Spectrometry (HJ 1315-2023) [20]. Available boron (B) was measured according to the Determination of Available Boron in Soil—Inductively Coupled Plasma Mass Spectrometry (DB12/T 1022-2022) [21]. The principle for measuring B content is as follows: After extraction, the sample is analyzed using inductively coupled plasma mass spectrometry (ICP-MS). Qualitative analysis is conducted based on the specific mass-to-charge ratio of the element. External standard calibration is employed, where the ratio of the mass spectrometry signal intensity of the element of interest to the internal standard element’s signal intensity is directly proportional to the concentration of the element under investigation. This method is used for the determination of boron content in soil samples. Germanium (Ge) content was analyzed using Analysis Methods for Regional Geochemical Samples—Part 16: Determination of Germanium Contents by Inductively Coupled Plasma Mass Spectrometry (DZ/T 0279.16—2016) [22]. The principle for measuring Ge is as follows: The sample is dissolved in nitric acid and made to a known volume, then mixed thoroughly. The solution to be analyzed is introduced into the radio-frequency plasma through pneumatic nebulization. Using a quadrupole ICP-MS, germanium ions are separated based on their mass-to-charge ratio. Detection is performed using a detector, and the Ge content is quantitatively analyzed using a calibration curve method. This method is applied for the determination of Ge content in soil samples. Iodine (I) content was measured following Soil and Stream Sediment—Determination of Iodine Content—Pressurized Ammonia Extraction Inductively Coupled Plasma Mass Spectrometry (GB/T 42333-2023) [23]. The sample is heated with dilute ammonia solution in a sealed digestion vessel to extract iodine. The iodine content in the sample solution is then measured using ICP-MS. Iodine isotopes are identified for qualitative analysis, and, within a certain concentration range, the ICP-MS count of iodine isotopes is directly proportional to the mass concentration of iodine. The iodine content in the sample is calculated by measuring the ICP-MS counts of iodine isotopes. Mercury (Hg), selenium (Se), and arsenic (As) were determined using the method described in Soil and Sediment—Determination of Mercury, Arsenic, Selenium, Bismuth, and Antimony—Microwave Digestion/Atomic Fluorescence Spectrometry (HJ 680-2013) [24], with an Atomic Fluorescence Spectrometer.

Total fluoride (total F) was analyzed using the Soil—Determination of Water-Soluble Fluoride and Total Fluoride—Ion Selective Electrode Method (HJ 873-2017) with a Fluoride Ion Composite Electrode (PF-202-L, INASE Scientific Instrument Co., Ltd., Shanghai, China) [25]. Total phosphorus (total P) and available phosphorus (available P) were measured using Inductively Coupled Plasma Emission Spectroscopy. Total nitrogen (total N) and hydrolyzable nitrogen (hydrolyzable N) were determined using a Kjeldahl Nitrogen Analyzer. Total potassium (total K) and exchangeable potassium (exchangeable K) were also measured using the Kjeldahl method. Organic matter was determined using the combustion–oxidation method with non-dispersive infrared detection.

To ensure high accuracy, all determination procedures are completed in professional laboratories and in accordance with published standards. Certified reference materials (HJ/T 166–2004 and DZ/T 295-2016) were used to verify the analytical precision, and the relative standard deviation for all samples was kept below 5%.

### 2.3. Potential Ecological Risk Index

The potential ecological risk index (PERI), originally proposed by Hakanson, was employed to evaluate the integrated ecological hazards posed by heavy metals in sludge. This method quantifies both the contamination level and toxicological effects of metals through two components: the contamination factor ($C_{f}^{i}$) and the toxic response factor ($T_{r}^{i}$) [26,27]. The contamination factor for each metal was calculated as follows:

(1) $$C_{f}^{i}=\frac{C_{i}}{C_{b}^{i}}$$

where $C_{i}$ represents the measured concentration of metal i, and $C_{b}^{i}$ denotes its reference background value. The toxic response factors ($T_{r}^{i}$) were assigned based on metal-specific ecotoxicity: Cd (30), Hg (40), As (10), Pb (5), Cu (5), Cr (2), Zn (1), and Ni (5) [28].

The single-metal ecological risk factor ($E_{r}^{i}$) was computed as follows:

(2) $$E_{r}^{i}=T_{r}^{i}\times C_{f}^{i}$$

The comprehensive ecological risk index (*RI*) was derived by summing the $E_{r}^{i}$ values for all metals:

(3) $$RI=\sum E_{r}^{i}$$

Risk levels were categorized as follows: low (RI<150), moderate (150≤RI<300), considerable (300≤RI<600), high (600≤RI<1200), and extreme (RI≥1200). This approach enables a systematic evaluation of multi-metal synergistic effects, prioritizing metals with higher toxicity and bioavailability. Reference values were adopted from regional soil quality guidelines to ensure context-specific relevance [29].

### 2.4. Pollution Load Index

The Pollution Load Index (PLI) was applied to evaluate the cumulative contamination of heavy metals in sediments by integrating individual contamination factors ($C_{i}/C_{b}^{i}$) into a unified metric [30,31]. The PLI, calculated as the geometric mean of contamination factors values for all analyzed metals, is expressed as follows:

(4) $$PLI={\left(\prod_{i=1}^{n}\frac{C_{i}}{C_{b}^{i}}\right)}^{1/n}$$

where $C_{i}$ represents the measured concentration of metal i, $C_{b}^{i}$ denotes its reference background value, and n represents the total number of metals. The interpretation of PLI values follows a standardized classification: values below 1 indicate no pollution, 1–2 suggest moderate pollution, 2–3 reflect high pollution, and values exceeding 3 signify severe pollution [32,33]. This index synthesizes multi-element data to assess spatial and temporal variations in sediment quality, with background values derived from regional geochemical baselines to ensure contextual accuracy.

### 2.5. Support Vector Machine (SVM) Model

Support Vector Machine (SVM) is a robust and widely used supervised classification technique based on the concept of maximum margin separation in the feature space. The core idea behind SVM is to find an optimal hyperplane that maximizes the margin between different classes in the dataset [34].

SVM operates by mapping the input features into a high-dimensional space through a kernel function, where the decision boundary is determined. The most commonly applied kernel for SVM is the Radial Basis Function (RBF) kernel, which measures the similarity between data points. This kernel allows the model to handle non-linear relationships between features and outputs effectively [35,36].

### 2.6. Decision Tree and Random Forestx

The Decision Tree (DT) and Random Forest (RF) models were employed for classification due to their strong performance in handling both categorical and continuous data, as well as their robustness in dealing with missing values and overfitting. DT C5.0 is an extension of the classical decision tree algorithm, designed to generate a set of decision rules that split the data at each node based on the feature that maximizes information gain [37,38]. Where information gain is calculated using entropy:

(5) $$Gain(S,A)=Entropy(S)-\sum_{v\in Values(A)}\frac{\left|S_{v}\right|}{\left|S\right|}Entropy(S_{v})$$

where *S* is the dataset, *A* is a candidate feature, $S_{v}$ denotes subsets split by *A*, and entropy measures class impurity:

(6) $$Entropy(S)=-\sum_{i=1}^{n}p_{i}log_{2}p_{i},$$

with $p_{i}$ representing the proportion of class i in *S*. DT C5.0 enhances robustness through post-pruning and rule-based simplification, addressing overfitting while maintaining interpretability for environmental classification tasks.

The Random Forest model is an ensemble method that constructs multiple decision trees, each trained on a random subset of the data. The final prediction is made by averaging the predictions of all the trees or taking a majority vote [39]. The prediction for a given input *X* in Random Forest can be written as follows:

(7) $$\hat{y}=\underset{c}{argmax}\sum_{k=1}^{K}f\left(X\right)$$

where *K* is the total number of trees, and $f\left(X\right)$ is an indicator function. This approach reduces variance and improves generalization by decorrelating individual tree errors, making it suitable for high-dimensional or noisy datasets.

### 2.7. Extreme Gradient Boosting (XGBoost)

The eXtreme Gradient Boosting (XGBoost) algorithm was employed as a state-of-the-art ensemble learning method, offering distinct advantages over traditional machine learning techniques such as support vector machines and random forests. Unlike conventional approaches, XGBoost integrates gradient boosting with advanced regularization mechanisms, enabling superior handling of complex, non-linear relationships while mitigating overfitting. Its computational efficiency, scalability, and ability to process sparse data make it particularly suitable for high-dimensional datasets [40].

XGBoost is an implementation of gradient boosting, where each tree in the model is trained to minimize a loss function using gradient descent techniques. What sets XGBoost apart is its incorporation of regularization terms in the objective function, which helps to prevent overfitting and ensures that the model generalizes well to unseen data. This feature is essential when dealing with large, complex datasets where overfitting is a common issue [41,42]. Additionally, XGBoost employs advanced techniques, such as tree pruning and parallel processing, to optimize training time, making it highly efficient for both large-scale and small-scale applications [43,44]. The model’s objective function integrates both prediction accuracy and structural complexity:

(8) $$Obj(\phi )=\sum_{i=1}^{n}L(y_{i},{\hat{y}}_{i})+\sum_{k=1}^{K}\Omega (f_{k})$$

where $L(y_{i},{\hat{y}}_{i})$ quantifies the discrepancy between observed $y_{i}$ and predicted ${\hat{y}}_{i}$ values, and $\Omega (f_{k})$ penalizes model complexity through:

(9) $$\Omega (f_{k})=\gamma T+\frac{1}{2}\lambda ∥w{∥}^{2}$$

where, *γ* and *λ* are regularization parameters controlling the penalty for leaf count (*T*) and weight magnitude (*w*), respectively.

### 2.8. Synthetic Minority Over-Sampling Technique

To address the class imbalance inherent in the dataset, the Synthetic Minority Over-Sampling Technique (SMOTE) was employed to enhance the representation of minority classes. SMOTE operates by generating synthetic samples for underrepresented categories through linear interpolation between existing minority class instances and their k-nearest neighbors [45,46]. For each minority observation $x_{i}$, a synthetic sample $x_{new}$ is generated along the vector connecting $x_{i}$ to a randomly chosen neighbor $x_{j}$. The algorithm proceeds as follows:

(10) $$x_{new}=x_{i}+\lambda \cdot (x_{j}-x_{i})$$

where λ denotes a random interpolation coefficient. This process effectively expands the decision boundary region for minority classes while preserving the intrinsic data distribution characteristics. The oversampling ratio was calibrated to achieve class equilibrium based on preliminary analysis of the original class distribution. SMOTE implementation excluded the majority of class samples to prevent artificial distortion of natural data patterns, ensuring the synthetic instances maintained feature space consistency with genuine observations.

### 2.9. Evaluation of Predictive Performance

The performance of the classification models was rigorously assessed using established evaluation metrics, including accuracy, precision, recall, and area under the Receiver Operating Characteristic (ROC). These metrics were selected to comprehensively evaluate the model’s discriminative power, calibration, and robustness [47,48,49].

Accuracy quantifies the proportion of correctly classified instances relative to the total samples and is defined as follows:

(11) $$Accuracy=\frac{TP+TN}{TP+TN+FP+FN}$$

where TP, TN, FP, and FN represent true positives, true negatives, false positives, and false negatives, respectively. While accuracy provides an intuitive measure of overall performance, it may be biased in imbalanced datasets.

Precision measures the reliability of positive predictions, calculated as follows:

(12) $$Precision=\frac{TP}{TP+FP}.$$

Recall evaluates the model’s ability to identify all relevant positive instances:

(13) $$Recall=\frac{TP}{TP+FN}.$$

The receiver operating characteristic (ROC) curve visualizes the trade-off between the true positive rate and the false positive rate across varying classification thresholds. The AUC-ROC summarizes this curve into a scalar value, representing the probability that the model ranks a random positive instance higher than a negative one. An AUC of 1.0 indicates perfect discrimination, while 0.5 suggests random guessing.

### 2.10. SHapley Additive exPlanations Interpretation Frameworkx

SHAP (SHapley Additive exPlanations) is a widely used method for model interpretability, especially in complex machine learning models. It is grounded in cooperative game theory and provides a way to assign each feature a contribution value based on its impact on the model’s output. The SHAP value for each feature reflects its marginal contribution when added to a subset of features, enabling a more transparent understanding of the model’s predictions. SHAP provides both global and local interpretability, where global refers to the general importance of each feature across the entire model, and local focuses on the contribution of features to a specific prediction [50,51].

For our analysis, SHAP values were calculated to assess the contribution of each feature to the overall prediction of environmental factors. This allows for a deeper understanding of the interaction effects between features, providing more interpretability to the predictive models used in our study.

## 3. Results and Discussions

### 3.1. Statistical Analysis of Soil Sample Characteristics

Table 1 presents the statistical characteristics of soil pollutant content in the Nansi Lake region. The metal elements involved in the evaluation of PERI and PLI are arsenic (As), cadmium (Cd), chromium (Cr), copper (Cu), mercury (Hg), nickel (Ni), lead (Pb), and zinc (Zn). Comparison with the background values of Jining City reveals that, except for Cr, the average concentrations of the remaining heavy metals are higher than the background values. The average concentrations of As, Hg, Cd, Cu, Pb, Zn, and Ni are 1.22, 1.67, 1.14, 1.09, 1.13, 1.18, and 1.15 times higher than the background values, respectively. Additionally, comparison with the Soil Environmental Quality—Risk Control Standard for Soil Contamination of Agricultural Land (https://www.mee.gov.cn/ywgz/fgbz/bz/bzwb/trhj/201807/W020190626595212456114.pdf, accessed on 18 October 2024) shows that the concentrations of all heavy metals are within the recommended standard limits. This indicates that, due to human activities, soil in the study area has begun to exhibit varying degrees of heavy metal accumulation, and immediate countermeasures should be taken to prevent the further spread of contamination. From the coefficient of variation analysis, it is evident that Hg and Cd exhibit high variability, with coefficients of variation of 107% and 43%, respectively. The kurtosis and skewness of Hg and Ni are extremely high, with substantial positive skewness, indicating that extremely high values exist for Hg and Ni in certain samples. This suggests that the concentrations of Hg, Cd, and Ni in the soil are strongly influenced by human activities, leading to spatial aggregation, with significant regional differences. The local soil is slightly alkaline, and, for certain pollution indicators, the average concentration exceeds the background values of Jining City, highlighting the phenomenon of pollution accumulation. The elements selenium (Se), manganese (Mn), boron (B), germanium (Ge), molybdenum (Mo), exchangeable potassium (K), total phosphorus (P), and available phosphorus (P) show high kurtosis with positive skewness, indicating that these pollution indicators are subject to accumulation in certain areas.

The Nansi Lake region is known for its well-developed fisheries, and rice is cultivated along the southwestern shore of the lake. With relatively high rainfall, the area experiences significant soil erosion and leaching. The accumulation of phosphorus in the soil can lead to phosphorus loss, which increases the potential for regional source pollution in the lake. This could negatively impact the local fisheries in the future.

### 3.2. Spatial Distribution of Soil PLI and PERI

**Figure 2:**
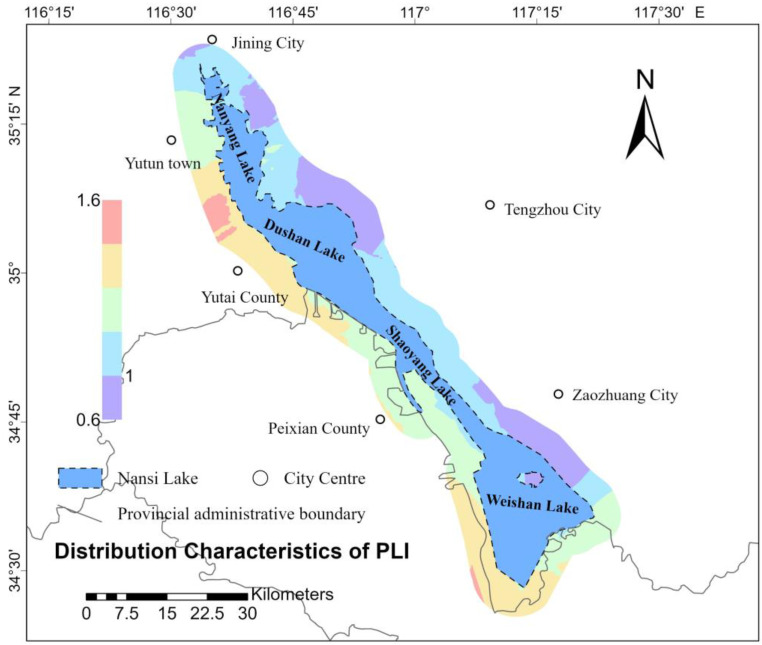
Spatial distribution characteristics of soils PLI in the study area.

**Figure 3:**
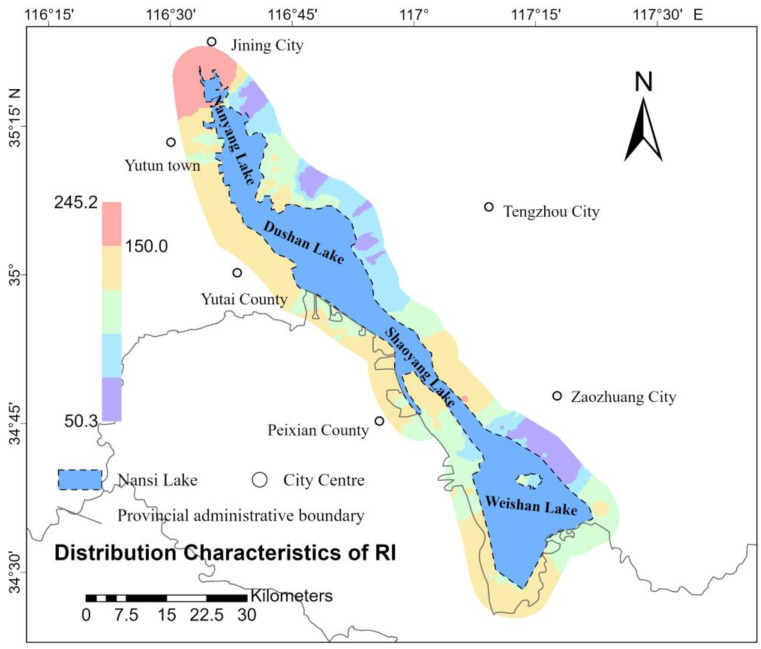
Spatial distribution characteristics of soils RI in the study area.

Spatial interpolation involves using the known values of sample points and their spatial locations to estimate the values of unknown points, thereby transforming discrete measurement data into a continuous surface. By applying spatial interpolation to the soil PLI and the PERI at various sample points, it is possible to visually represent the distribution of these indices across the study area. This method allows for a clearer understanding of the spatial variation in the PLI and PERI values within the research scope. In this study, ArcGIS Pro 3.3.2 software was used; spatial distribution characteristics maps of the PLI (Figure 2) and RI (Figure 3) for eight heavy metals in the soil of the study area were generated using the Empirical Bayesian Kriging (EBK) interpolation technique. Figure 2 illustrates that the high-value areas of the PLI in the study region are predominantly concentrated around Yutai County, located near the western shore of Nansi Lake, as well as along the southern shore of the lake. This distribution may be related to the development of agriculture and aquaculture in these regions. Figure 3 indicates that the high-value areas of the PLI are mainly concentrated in the northernmost part of Nansi Lake, around the city center of Jining, and in the central part of Nansi Lake, specifically, in Weishan County. This distribution may be associated with industrial production and tourism-related transportation in these areas.

### 3.3. Training and Selection of Machine Learning Classification Models

Based on the statistical characteristics of pollutant content, PLI analysis, and PERI analysis, we perform a horizontal comparison and use multiple machine learning models to predict soil quality. Additionally, we employ the SHAP model to identify key pollution indicators and analyze their impacts.

We categorize the sample PLI evaluation results into two groups: “no PLI pollution” (PLI < 1) and “moderate PLI pollution” (PLI between 1 and 2), with 58 and 122 samples in these categories, respectively. The sample PERI evaluation results are divided into “low RI risk” (RI < 150) and “moderate RI risk” (RI between 150 and 300), with 151 and 28 samples in these categories, respectively. Based on the PLI and PERI evaluation results, a new assessment method is created. Samples classified as “no PLI pollution” and “low RI risk” are rated as Class0—No Risk, those classified as “no PLI pollution” and “moderate RI risk” or “moderate PLI pollution” and “low RI risk” are rated as Class1—Low Risk, and those classified as “moderate PLI pollution” and “moderate RI risk” are rated as Class2—High Risk. The sample sizes for Class0, Class1, and Class2 are 56, 97, and 27, respectively. Once a new assessment technique was set up, we implemented SMOTE oversampling in order to equalize sample sizes across each class for further classification training using machine learning. After reconciling the previously mentioned data, all classes have an equal quantity of 97 samples which shows that in total 291 samples will be utilized in the posteriori model training.

**Figure 4:**
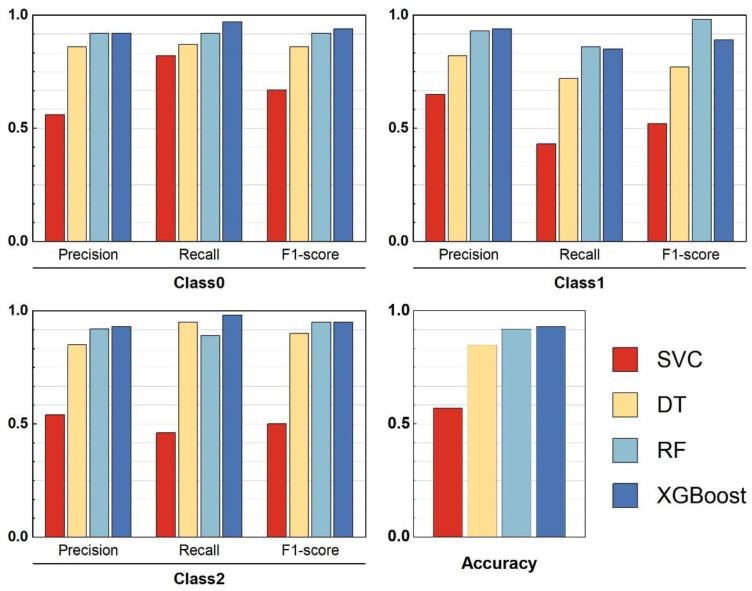
Evaluation metrics for machine learning classification models.

XGBoost may be well known for its ability to work with large datasets, but it is just as effective with smaller datasets when accompanied by adequate model tuning and validation strategies. In order to improve the performance of Random Forest and XGBoost, we applied for 5-fold cross-validation alongside Random Search to better optimize the model hyperparameters. Even though our sample size was small, these methods greatly reduce overfitting while increasing model performance. We utilized the SVC, RandomForestClassifier, and DecisionTreeClassifier functions from the scikit-learn library (version 1.6.1) in Python (version 3.10.11) to train the SVC, RF, and DT models, and the RandomizedSearchCV function to find the most suitable hyperparameters for RF and XGBoost. The XGBClassifier function from the xgboost library (version 2.1.4) in Python was employed to train the XGBoost model. The SHAP library (version 0.44.1) in Python was used to provide visual explanations for the machine-learning models. The four machine learning classifiers were trained using new data from 291 samples, and, after tuning the parameters of each model, the classification model evaluation metrics were obtained, as shown in Figure 4.

**Figure 5:**
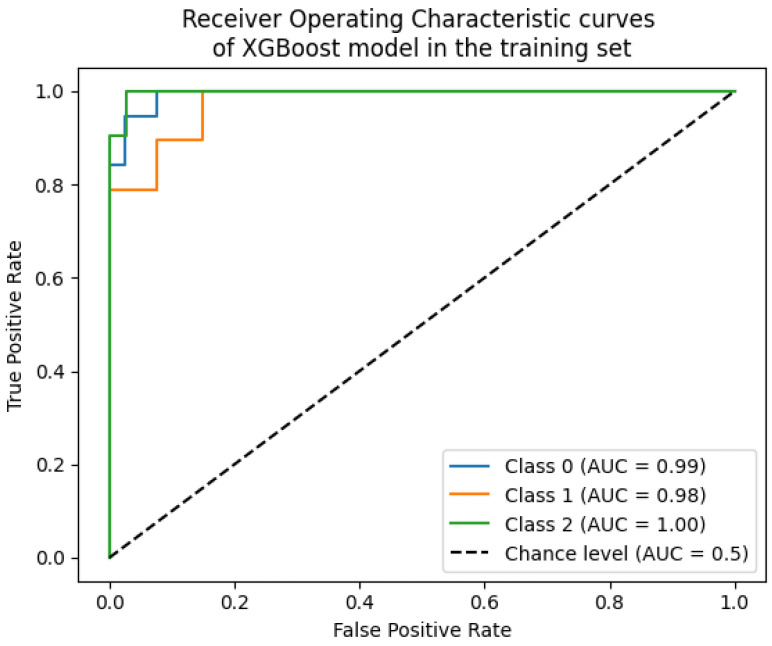
Receiver operating characteristic curves of the XGBoost model in the training set.

A comparison from Figure 4 reveals that the RF and XGBoost models exhibit the best classification performance, with higher precision and recall. Additionally, the training performance across different classes is relatively stable. XGBoost slightly outperforms RF in terms of accuracy, with values of 0.93 and 0.92, respectively. Therefore, the XGBoost model was selected to construct the classification model, with 20% of the samples used as the test set. Figure 5 displays the ROC curve of the model.

ROC, as well as Area Under the Curve (AUC), are commonly used metrics to determine the effectiveness of a classifier. The closer the AUC value is to 1, the better the performance of the classifier. Similarly, an AUC value which is closer to 0 indicates poorer performance. In this scenario, the XGBoost classifier is better at predicting data. Then, the prediction result explanation will be performed through the use of SHAP value.

It is a common belief that XGBoost is only suitable for bigger datasets, however, it is feasible to use this algorithm on smaller datasets according to our research results. Also, based on our data, XGBoost outperforms Random Forest in terms of prediction accuracy. This finding shows that even what is considered a small sample can yield useful results because of the flexibility and power of XGBoost. It can deal with huge data sets, which proves the ability of this algorithm even apart from this study.

### 3.4. Explanatory Analysis Based on SHAP

**Figure 6:**
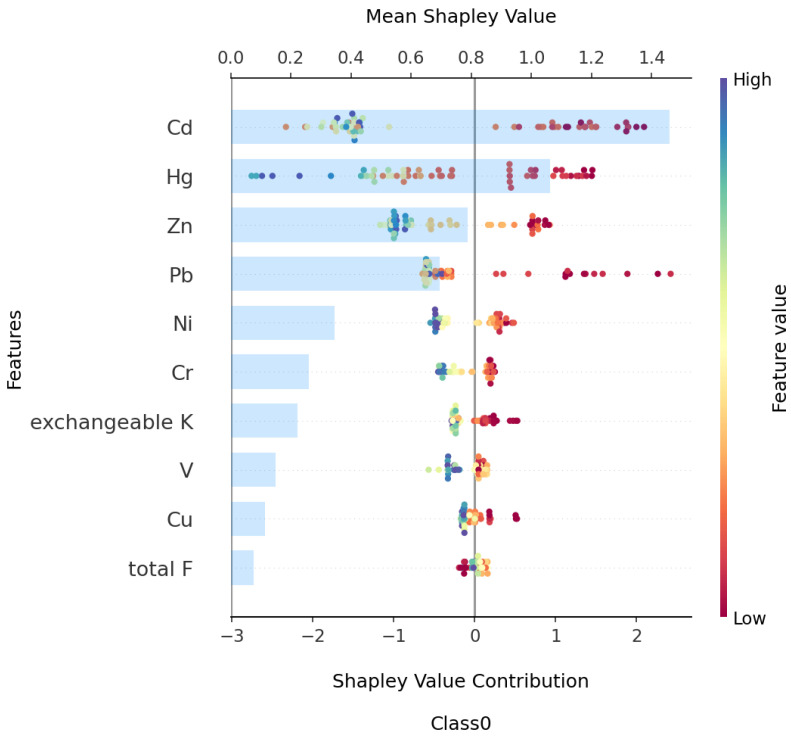
Complex beeswarm plot of SHAP contribution for Class0 top 10 features.

**Figure 7:**
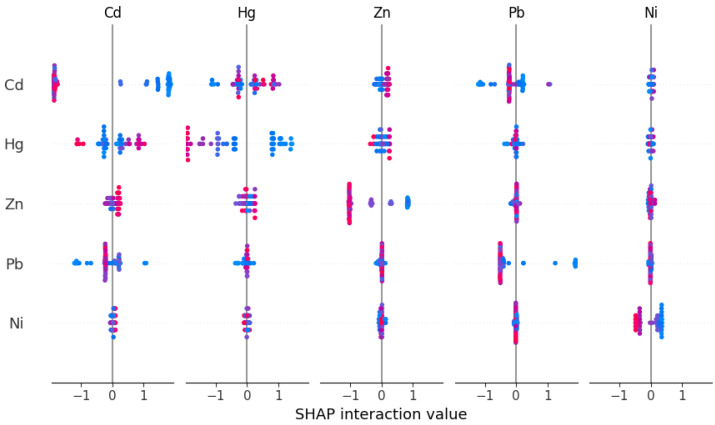
Interactive scatter plot of SHAP for Class0 top five features.

**Figure 8:**
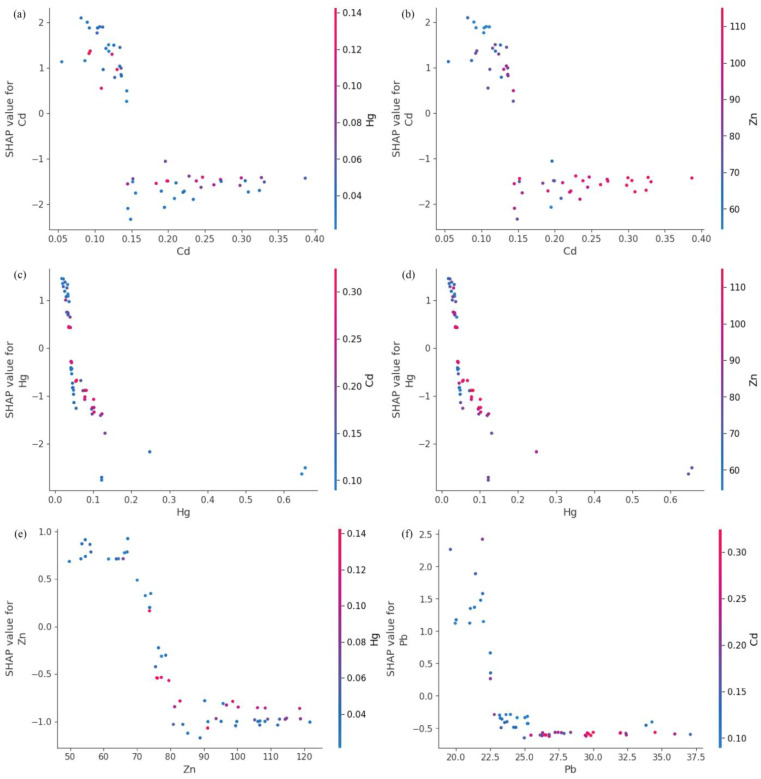
Dependence plot of SHAP for Class0 high-contribution features. (**a**) Hg on action of Cd. (**b**) Zn on action of Cd. (**c**) Cd on action of Hg. (**d**) Zn on action of Hg. (**e**) Hg on action of Zn. (**f**) Cd on action of Pb.

To generate Figure 6 and the subsequent figures, we utilized SHAP values to evaluate feature importance. SHAP values provide a method to explain the contribution of each feature to the model’s predictions. In each sample, we computed the SHAP values associated with each characteristic, representing the impact of the characteristic on the prediction in relation to its impact on the average prediction. These values were then plotted in the form of scatter plots, where the position of each point represents the SHAP value of a specific feature for a given sample. A higher mean SHAP value indicates greater importance of that feature in driving the model’s prediction, and the mean Shapley value of the feature is also displayed behind the scatter points, which is a blue histogram. Based on Figure 6, the top ten features contributing to Class0—No Risk, ranked from highest to lowest, are Cd, Hg, Zn, Pb, Ni, Cr, exchangeable K, V, Cu, and total, with their mean absolute SHAP values being 1.46, 1.06, 0.78, 0.69, 0.34, 0.25, 0.22, 0.14, 0.11, and 0.07, respectively. Interactive scatter plots (Figure 7) and dependence plots (Figure 8) were created to analyze the interactions between high-contributing features and the impact of these features on the model’s predictions. Figure 7 demonstrates the influence of interactions between features on model predictions. Each row and column in the figure corresponds to a feature, with the cells depicting the interactions between pairs of features. The color represents the strength of the interaction between features. Red dots indicate high interaction, while blue dots indicate low interaction. In Figure 8, each cell can exhibit both the dependency between two features and the Shapley values associated with various values of a single feature. The horizontal axis indicates feature values, while the left vertical axis signifies Shapley values. The scatter points represent individual samples, with a redder color indicating a higher value of the interacting feature for that particular sample. From the figures, it is evident that the concentrations of Cd, Hg, Zn, and Pb pollution features exhibit a trend of “decreasing to stable” as their values increase, showing negative contributions to Class0. Since Class0 represents regions predicted to have no pollution risk, this suggests that, under the current pollution evaluation standards, higher concentrations of these pollution features increase the likelihood of a sample point being classified outside of Class0, indicating a higher potential pollution risk.

**Figure 9:**
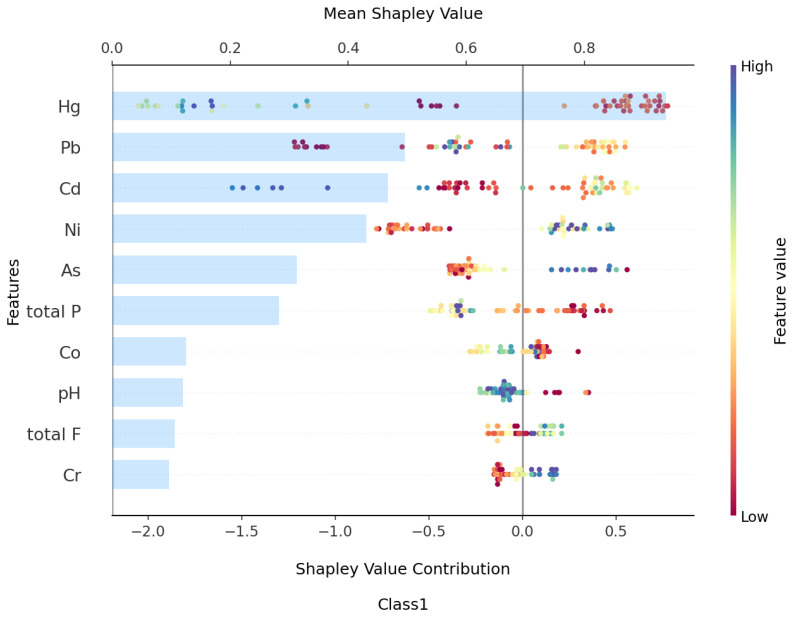
Complex beeswarm plot of SHAP contribution for Class1 top 10 features.

**Figure 10:**
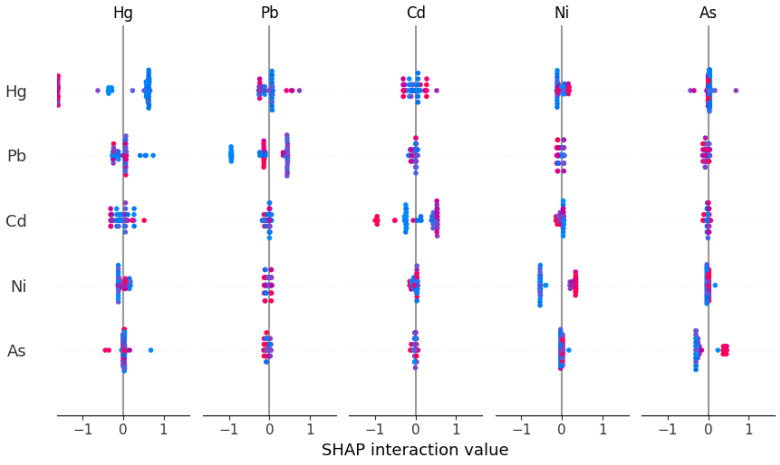
Interactive scatter plot of SHAP for Class1 top five features.

**Figure 11:**
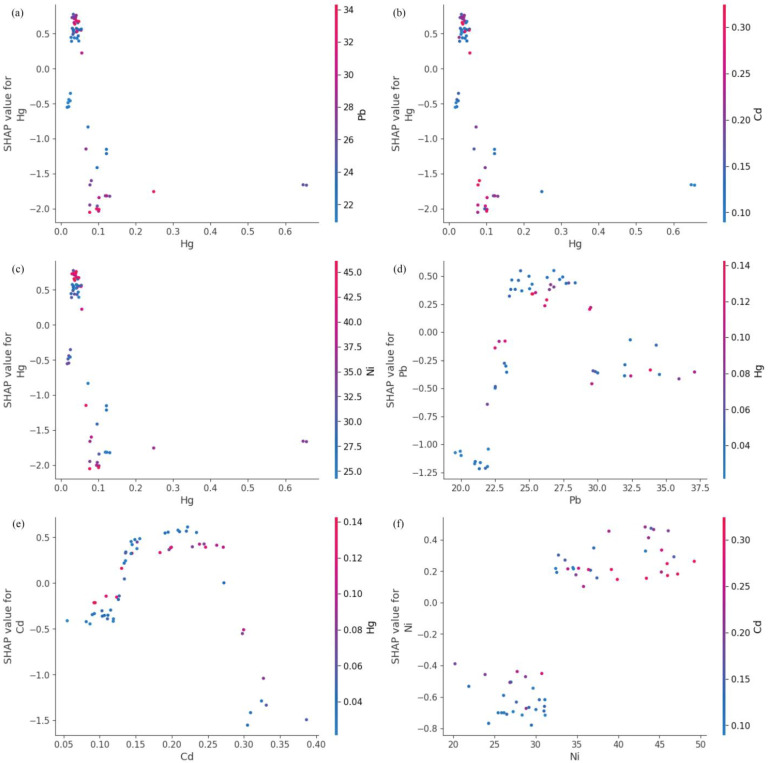
Dependence plot of SHAP for Class1 high-contribution features. (**a**) Pb on action of Hg. (**b**) Cd on action of Hg. (**c**) Ni on action of Hg. (**d**) Hg on action of Pb. (**e**) Hg on action of Cd. (**f**) Cd on action of Ni.

Based on Figure 9, the top ten features contributing to Class1—Low Risk, ranked from highest to lowest, are Hg, Pb, Cd, Ni, As, total P, Co, pH, total F, and Cr, with their mean absolute SHAP values being 0.93, 0.49, 0.46, 0.43, 0.31, 0.28, 0.12, 0.11, 0.10, and 0.09, respectively. From Figure 10 and Figure 11, it can be observed that the Hg pollution feature shows a “decrease then stabilize” trend as its concentration increases, indicating a negative contribution to Class1. On the other hand, the concentrations of Hg pollution features exhibit an “increase then stabilize” trend, reflecting a positive contribution to Class1. Both Cd and Pb pollution features follow a “rise then fall” pattern as their concentration increases. The contribution of Cd to Class 1 is maximized when its concentration reaches around 0.2, with a higher contribution when the Hg concentration is lower. Similarly, Pb contributes the most to Class1 when its concentration reaches around 26, and, like Cd, its contribution increases as Hg concentration decreases. Based on the SHAP explanation results for Class1, under the current pollution evaluation method, the concentration of Ni pollution features may have strong control over the occurrence of Low Risk, while the accumulation of pollutants such as Hg, Pb, and Cd could further increase the pollution risk. Studies have shown that local Ni enrichment is mainly controlled by soil parent materials and natural processes, followed by pollution from transportation [53]. This reinforces the point that the supplementation of Ni in the soils of the Nansi Lake basin from human activities is yet in the quite early stages. Therefore, the government would need to implement policy approaches that limit further tourism-induced Ni pollution. At the same time, Figure 9 shows that soil samples with higher pH values, indicating a weakly alkaline soil, exhibit a negative contribution to the risk of this type of pollution in the soil due to their higher pH values. Research indicates that elevated soil pH can decrease the available concentration of heavy metals in soil solutions, as increased pH facilitates the adsorption of heavy metal ions by soil colloid particles, subsequently minimizing the uptake of heavy metals by crops [54,55]. Considering the relatively low mean Shapley Values of pH in Class0 and Class2, it is plausible to infer a negative correlation between pH and the risk of soil contamination. However, the impact of this correlation is not strong.

**Figure 12:**
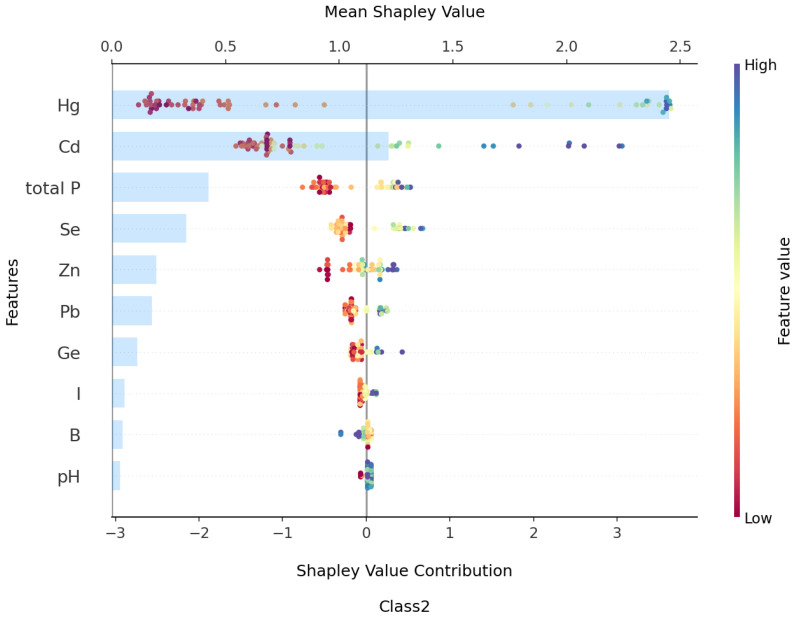
Complex beeswarm plot of SHAP contribution for Class2 top 10 features.

**Figure 13:**
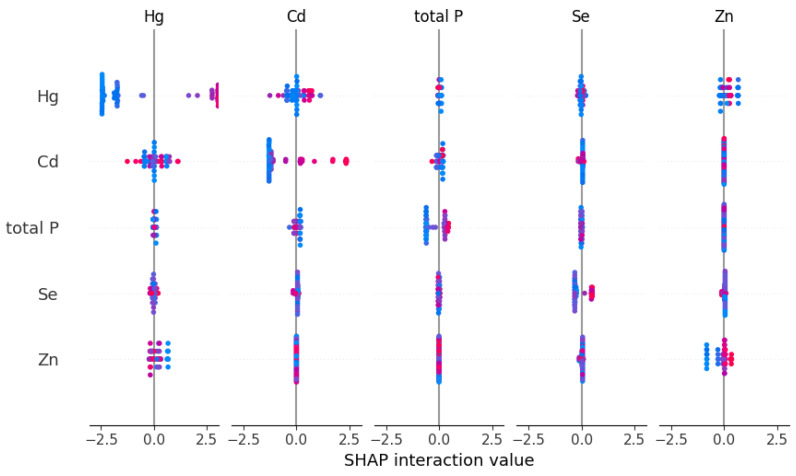
Interactive scatter plot of SHAP for Class2 top five features.

**Figure 14:**
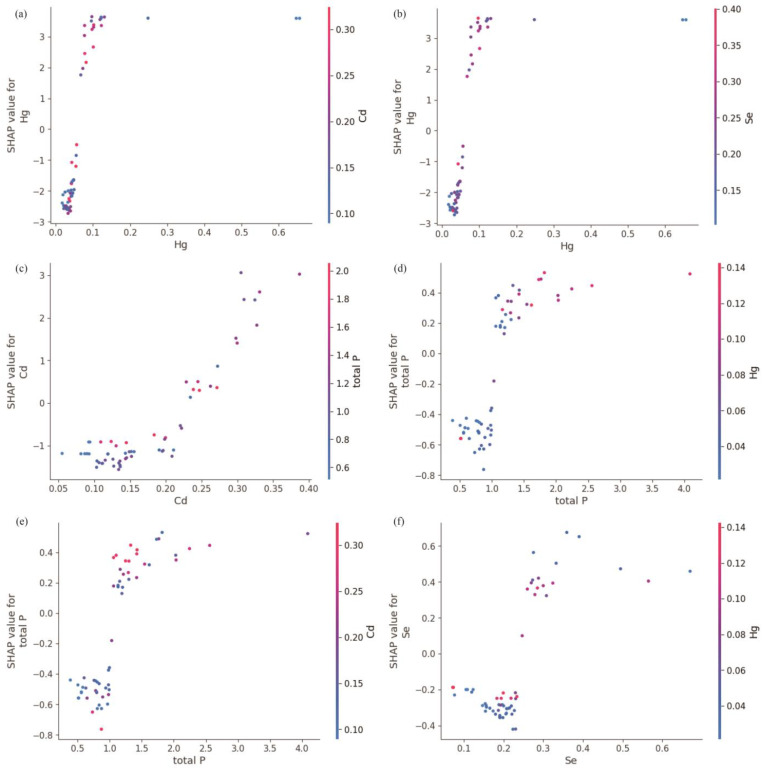
Dependence plot of SHAP for Class2 high-contribution features. (**a**) Cd on action of Hg. (**b**) Se on action of Hg. (**c**) total P on action of Cd. (**d**) Hg on action of total P. (**e**) Cd on action of total P. (**f**) Hg on action of Se.

Based on Figure 12, the top ten features contributing to Class2—High Risk, ranked from highest to lowest, are Hg, Cd, total P, Se, Zn, Pb, Ge, I, B, and pH, with their mean absolute SHAP values being 2.45, 1.21, 0.42, 0.32, 0.19, 0.17, 0.11, 0.05, 0.04, and 0.03, respectively. From Figure 13 and Figure 14, it can be observed that the pollution features of Hg, Cd, total P, and Se show an “increase then stabilize” trend as their concentrations rise, indicating a positive contribution to Class2. Additionally, as Hg and Cd concentrations increase, the contribution of total P to Class2 also increases, suggesting a positive correlation between these pollution features and the occurrence of pollution risk.

In conjunction with the SHAP analysis results for Class0 and Class1, under the current pollution evaluation method, even the accumulation of small amounts of Hg, Cd, total P, and Pb can result in significant pollution risks. This is in contrast to the slower accumulation observed with other pollution features, such as Zn, Pb, and Ni. Furthermore, based on statistical data, although As, Cu, Co, B, I, V, Mo, and K are accumulated in the soil samples, they have a relatively small impact on the generation of pollution risk. The pollution features Mn, N, and organic matter do not exhibit significant effects under the current evaluation method, indicating that further research may be required.

## 4. Conclusions

The soil pollution in the Nansi Lake region was initially evaluated using three traditional methods: statistical characteristic analysis, PLI, and PERI. The statistical analysis indicated elevated concentrations of As, Hg, Cd, Cu, Pb, Zn, and Ni, with Hg and Cd showing significant variability. Furthermore, Hg and Ni demonstrated extremely high kurtosis and skewness, with significant positive skewness. Similarly, Se, Mn, B, Ge, Mo, exchangeable K, total P, and available P also showed high kurtosis and positive skewness. The spatial analysis of PLI and PERI indicated that the high-value areas of PLI were primarily located around Yutai County near the western shore of Nansi Lake and the southern shore of Nansi Lake. The high-value areas of PERI were concentrated in the northernmost part of Nansi Lake, around the city center of Jining, and in the central part of Nansi Lake, particularly in Weishan County.

The PLI and PERI evaluations were integrated to establish new soil quality classification standards. The classification models, including SVC, DTC 5.0, Random Forest (RF), and XGBoost, were trained using these standards. XGBoost demonstrated superior performance, achieving 93% accuracy, highlighting its suitability for both large and smaller datasets.

XGBoost’s interpretability was enhanced through SHAP analysis, which identified key pollutants influencing soil quality classification. Through SHAP, we observed that Cd, Hg, and Zn were the top contributors to the Class0—no risk, showing that, under current pollution evaluation standards, higher concentrations of these pollutants were associated with lower risk classification due to their inverse relationships with soil health indicators. These pollution characteristics contribute significantly to Class0 when they are at low values, indicating that an increase in the concentration of these pollution characteristics will lead to pollution risks in the sample. The analysis also reveals that Hg and Pb pollutants significantly contribute to the Class1—low-risk. Furthermore, the concentration of Ni pollution characteristics may have a strong controlling effect on the occurrence of low risk, while the accumulation of pollutants such as Hg, Pb, and Cd may further increase pollution risks.

For the Class2—high risk, SHAP analysis highlighted the pivotal role of pollutants such as Hg, Cd, total P, and Se. The increasing concentrations of these elements were strongly correlated with a higher likelihood of classifying soil as high-risk. These findings demonstrate the value of using advanced machine learning models like XGBoost to assess and predict soil pollution and provide a basis for developing more effective soil quality management strategies in the Nansi Lake region.

## Figures and Tables

**Table 1:** Statistical characterization of soil pollutant content.

	Min	Max	Mean	Standard Deviation	Coefficient of Variance	Kurtosis	Skewness	Background Value ^1^
As	3.75	25.65	12.70	3.60	0.28	−0.05	0.49	10.40
B	25.83	145.65	50.20	10.91	0.22	20.00	2.58	2.58
Cr	24.67	96.38	65.15	12.91	0.20	−0.65	0.18	66.00
Cd	0.04	0.41	0.18	0.08	0.43	−0.22	0.67	0.16
Cu	11.50	76.01	31.33	7.36	0.23	3.49	0.90	28.80
Co	6.98	24.19	12.98	2.65	0.20	0.95	0.67	0.67
Ge	1.06	3.51	1.56	0.34	0.22	9.29	2.73	1.33
Hg	0.01	0.66	0.06	0.07	1.07	46.95	6.00	0.04
I	0.47	6.44	2.23	1.13	0.51	0.39	0.88	0.88
Mn	353.04	2481.23	770.16	272.91	0.35	9.95	2.59	2.59
Mo	0.26	2.36	0.66	0.26	0.39	10.07	2.24	0.64
Ni	12.84	153.25	35.36	10.27	0.29	58.72	5.23	30.80
Pb	14.08	44.60	26.40	4.35	0.16	0.69	0.63	23.30
Se	0.03	1.24	0.25	0.17	0.68	12.75	3.20	3.20
V	42.56	116.67	80.43	15.11	0.19	−0.88	0.16	0.16
Zn	37.89	162.22	85.36	20.29	0.24	−0.50	0.13	72.20
total F	331.54	1372.39	661.08	158.61	0.24	0.72	0.60	-
total K	15.29	30.54	21.44	3.31	0.15	0.21	0.93	-
exchangeable K	62.00	1438.00	237.38	123.99	0.52	30.66	3.70	-
total N	0.26	3.85	1.56	0.67	0.43	0.17	0.75	-
hydrolyzable N	4.80	317.62	119.16	56.01	0.47	1.73	1.22	-
total P	0.35	4.09	1.12	0.56	0.50	8.41	2.27	-
available P	2.82	379.05	47.82	53.08	1.11	8.54	2.54	-

^1^ The background value is cited from references [52].

## Data Availability

Data will be made available on reasonable request from the corresponding author.
